# Catching Cold

**Published:** 1875-12

**Authors:** 


Catching Cold.—The season of the year will soon be upon us when people everywhere will be taking cold, and in many cases they will suffer much and die. A little care would often prevent it. In the first place, as one of the means to prevent a cold, the daily bath in a warm room, with much friction, is very important. In no case should the body be chilled. Use much friction over the chest and throat, and snuff into the nostrils a little of the water warmed to a comfortable temperature.

Next, after the bath, take daily exercise in the open air, neither too much nor too little; exposing the body somewhat to the cold and sun for a short time, but never exhausting it. One chief danger from colds is the exhausted state of the body that first occurs, so it is not able to resist unfavorable influences. People who are not very vigorous should avoid over exertion and keep the strength up to the highest point.

It will help those prone to colds to sleep all they can.

Another cause of colds is eating too heartily after a day's work, when there are not forces enough to digest the food and keep up the circulation. Eat moderately at night, if you would avoid a cold.

A cold in its early stages may be broken up by hot foot-baths, warmth to the body, especially a hot pack or a hot bath in the middle of the day, with much friction and quiet in a comfortable room. It is not advisable to take a hot bath at night in such cases.

When you have a cold don't eat much or work much unless you have great physical strength, when a hard day's work may be a good thing to equalize the circulation and restore the action of the skin, which always suffers when one takes cold.—*Herald of Health.*

